# A New Modulator of Neuroinflammation in Diabetic Retinopathy: USP25

**DOI:** 10.1007/s10753-024-01991-x

**Published:** 2024-03-04

**Authors:** Qiang Hu, Xue Zhang, Hongsong Peng, Jitian Guan, Zhangxin Huang, Bo Jiang, Dawei Sun

**Affiliations:** 1 Department of Ophthalmology, The Second Affiliated Hospital of Harbin Medical University, 157 Baojian Road, Harbin, 150086 China; 2 Future Medical Laboratory, The Second Affiliated Hospital of Harbin Medical University, Harbin, China

**Keywords:** diabetic retinopathy, ubiquitination, microglia, inflammatory response, ROCK/NF-κB signaling pathway.

## Abstract

Diabetic retinopathy (DR) is a diabetes-associated complication that poses a threat to vision, distinguished by persistent and mild inflammation of the retinal microvasculature. The activation of microglia plays a crucial role in driving this pathological progression. Previous investigations have demonstrated that ubiquitin-specific peptidase 25 (USP25), a deubiquitinating enzyme, is involved in the regulation of immune cell activity. Nevertheless, the precise mechanisms through which USP25 contributes to the development of DR remain incompletely elucidated. Firstly, we have demonstrated the potential mechanism by which ROCKs can facilitate microglial activation and augment the synthesis of inflammatory mediators through the modulation of NF-κB signaling pathways in a high-glucose milieu. Furthermore, our study has provided novel insights by demonstrating that the regulatory role of USP25 in the secretion of proinflammatory factors is mediated through the involvement of ROCK in modulating the expression of NF-κB and facilitating the nuclear translocation of the phosphatase NF-κB. This regulatory mechanism plays a crucial role in modulating the activation of microglial cells within a high-glycemic environment. Hence, USP25 emerges as a pivotal determinant for the inflammatory activation of microglial cells, and its inhibition exhibits a dual effect of promoting retinal neuron survival while suppressing the inflammatory response in the retina. In conclusion, the promotion of diabetic retinopathy (DR) progression by USP25 is attributed to its facilitation of microglial activation induced by high glucose levels, a process mediated by the ROCK pathway. These findings highlight the importance of considering USP25 as a potential therapeutic target for the management of diabetic neuroinflammation.

**Supplementary Information:**

The online version contains supplementary material available at 10.1007/s10753-024-01991-x.

## INTRODUCTION

Diabetic retinopathy (DR) stands as the predominant microvascular complication of diabetes mellitus (DM) and represents a chronic, progressive eye ailment that leads to irreversible vision impairment and even blindness in industrialized countries’ working-age populations [1, 2]. The pathological hallmarks of DR encompass inflammation, angiogenesis, and fibrosis [2]. Nevertheless, the exact mechanisms behind the development of DR, as well as the molecular and cellular processes that transpire during its early stages such as glial cell dysfunction, microvascular abnormalities, and neuronal degeneration, remain poorly understood. Recent studies have elucidated glial activation as the first event in the onset of DR [3]. Therefore, targeting glial cell dysfunction holds promising therapeutic potential for managing DR.

Acute inflammation functions as a physiological defense mechanism in response to injuries, whereas persistent inflammation has the potential to worsen damage and accelerate the development of diseases. Clinical studies have demonstrated samples of blood, aqueous humor, and vitreous humor taken from diabetic individuals showed an upregulation of the expression of pro-inflammatory growth factors, cytokines, and chemokines. These inflammatory mediators not only attract leukocytes to the vascular endothelium but also reactivate microglia during DR, leading to uncontrolled microglial proliferation and triggering retinal toxicity and neuronal cell death [4]. Microglia have a ramified shape under typical conditions, and they use their lengthy processes to survey the retina. However, they undergo activation and transform into amoeboids when the microenvironment is disturbed [5]. Inflammation is facilitated by the migration of amoeboid-microglia from the inner to the outer retina, during which they secrete proinflammatory markers including interleukin (IL)-1β, monocyte chemotactic protein-1 (MCP-1), and tumor necrosis factor-α (TNF-α) [6]. Inflammatory factors such as TNF-a can further activate retinal glial cells, disrupt the blood retinal barrier, and MCP-1 can attract more monocytes to infiltrate the retina, further exacerbating the development of DR. Glial reactivity (gliosis) in the retina is remarkably associated with early neuronal degeneration and vascular dysfunction, according to recent research [7]. Hence, understanding microglial dysfunction is paramount.

The ubiquitin-proteasome system (UPS) is a key component of the pathway associated with the degradation of proteins. UPS dysfunction results in the accumulation of neurotoxic proteins which is associated with a range of neurodegenerative disorders, such as retinitis pigmentosa, macular degeneration, glaucoma, and DR [8]. Through the analysis of Gene Expression Omnibus (GEO) datasets, we found a significant elevation in ubiquitination-related genes (URGs) in DR tissues compared to normal tissues, particularly ubiquitin-specific peptidase 25 (USP25). The ubiquitin-specific protease designated as USP25 is encoded by the USP25 gene, which is located on the human chromosome 21q11.2 region [9]. The elimination of the USP25 gene in a mouse model has demonstrated a reduction in neuroinflammation and the restoration of synaptic and cognitive function. Mechanistically, the deletion of USP25 attenuates microglia-mediated overproduction of pro-inflammatory cytokines. The inhibition of USP25 reestablishes homeostatic microglial signaling, thereby restoring synaptic and cognitive function in mice [10]. Furthermore, USP25 is established to have a crucial function in M1-like macrophage polarization and the pro-inflammatory response, which may be associated with pyruvate kinase muscle 2, a regulator of aerobic glycolysis and lactate production [11]. Although it has been widely documented that UPS-mediated protein degradation and neuroinflammation are both critically implicated in neurodegeneration, it is yet unknown how USP25 contributes to the pathogenesis of DR.

The activation of microglia in DR may yield both beneficial and detrimental outcomes [12]. While it aids in the removal of debris, which is beneficial, activated microglia can also inflict harm upon the normal retina, including its vascular endothelium, and trigger monocyte migration to retinal focal points, inciting a low-grade inflammatory response [13]. Thus, we hypothesized that under diabetic conditions, metabolic factors such as hyperglycemia and advanced glycation end products stimulate microglial activation, thereby fostering phagocytosis or pro-inflammatory element and inducing damage to normal retinal vascular endothelial cells and neurons, ultimately promoting inflammatory responses within the retina. We conjectured that USP25 may play a role in mediating this process.

## MATERIALS AND METHODS

### Bioinformatics Analysis

To investigate the role of URGs in DR, we initially retrieved the GSE111465 dataset of DR from the GEO database (https://www.ncbi.nlm.nih.gov/geo/). The ubiquitin and ubiquitin-like conjugation database (http://uucd.Biocuckoo.org) was searched to acquire the URGs. To ascertain the differential expression of the URGs between diseased and normal samples within the GSE11465 dataset, we utilized the R package “limma” and established the significance criteria for our analysis as adj *P* < 0.05 [14, 15]. Heatmap visualizations were employed to represent the differential gene expression data, and the differences in USP25 expression were illustrated using violin plots [15]. Subsequently, we conducted a Spearman correlation analysis to assess the correlation between USP25 and ROCK1/ROCK2.

### Animal Model of Diabetic Retinopathy and Intravitreal AAV-sh-USP25 Injection

Beijing Vital Rival Laboratory Animal Technology (Beijing, China) supplied the male C57BL/6 J mice (20–22 g in weight), which were allowed *ad libitum* access to food and water and provided a 12-h light/dark cycle environment. C57BL/6 J mice were randomly selected for the induction of diabetes. To induce diabetes, we injected male C57BL/6 J mice with 50 mg/kg of STZ intraperitoneally once daily for 5 consecutive days, while control (Ctrl) mice received an equivalent dose of citric acid buffer [16]. When the blood glucose levels of STZ-injected mice surpassed 16.7 mmol/L (300 mg/dL) for two consecutive days, these mice were deemed diabetic. Any mouse failing to develop diabetes was excluded from the experiment.

Four weeks following the injection of STZ, the right eye of mice with diabetes was intravitreally injected with AAV-sh-USP25 (3 μL; Hanbio Tech 80060228, Shanghai, China) (denoted as the “DM + AAV-sh-USP25” group in figures) or AAV-sh-Null (denoted as the “DM + AAV-sh-Null” group in figures). Meanwhile, the left eye was injected with an identical dose of normal saline (154 mmol/L NaCl) (denoted as the “DM” group). Age-matched normal Ctrl mice remained untreated. Mice were euthanized at 6, 8, or 12 weeks after the onset of diabetes.

### Cell Culture and Treatments

To investigate high glucose (HG)-induced USP25 expression in microglia, we used two distinct cell lines, HMC3 human microglia clone 3 and BV2 mouse microglia, both procured from Procell (Wuhan, China). These cell lines were grown at 37 °C in a humidified incubator with 5% CO2 in low-glucose (5 mmol/L) MEM supplemented with 10% (vol./vol.) FBS (catalog no. 10091148; Gibco, NY, USA) and 1% (vol./vol.) penicillin/streptomycin (catalog no. 15140122; Invitrogen, CA, USA). After 24 h, four groups of cells were established: normal Ctrl, HG treatment, HG + Si-USP25 treatment, and HG + negative control (NC) treatment.

To examine the impact of the ROCK/NF-κB signaling pathway on HG-induced microglial activation and the subsequent secretion of inflammatory factors, HMC3 cells were preincubated with the ROCK inhibitor Y-27632 (10 μmol/L; catalog no. HY-10071; MCE, USA) for a period ranging from 30 min to 1 h before their exposure to HG conditions.

### Immunofluorescence Analysis

For immunofluorescence labeling, 4% paraformaldehyde (PFA) was used to fix treated cells, cryosections, or paraffin sections for half an hour, followed by permeabilizing them with a 0.5% (vol./vol.) Triton X-100 solution in 1 × PBS. Subsequently, a blocking solution containing 1% (wt/vol.) BSA and 0.1% (vol./vol.) Triton X-100 in 1 × PBS was used. After a 1-h incubation and block at room temperature (RT), the sections were subjected to overnight incubation at 4 °C with the primary antibodies below: anti-ionized calcium-binding adaptor molecule 1 (Iba-1) antibody (1:500; catalog no. 019-19741; WAKO, Osaka, Japan); anti-pNF-κB antibody (Ser536) (1:500; catalog no. D14E12; CST, USA); and anti-USP25 antibody (1:50; catalog no. sc-398414; Santa Cruz, USA). Following three 10-min washes with 1 × PBS, donkey anti-mouse IgG H&L antibody (Alexa Fluor 488; 1:1000; catalog no. ab150105; Abcam, USA) or donkey anti-rabbit IgG H&L antibody (Alexa Fluor 647; 1:1000; catalog no. ab150075; Abcam) was introduced into the sections or cells, followed by incubation for 90 min at RT. After three additional 10-min washes with 1 × PBS, the DAPI-fluoromount-G (catalog no. 0100-20; Southern Biotech, AL, USA) was used to counterstain and mount the sections. Cells were counterstained with phalloidin (C2201S or C2207S; 1:100; Beyotime, China) for 1 h at RT. Finally, cells or sections were examined under a fluorescence microscope (Leica, Mannheim, Germany).

### Western Blotting

The retinal cells or tissues were lysed using ice-cold lysis buffer (Beyotime), which contained protease inhibitor cocktail (1:100; Beyotime) and PhosStop phosphatase inhibitors (1:10; Roche, Germany), along with subsequent centrifugation for 20 min at 12,000 rpm and 4 °C. The BCA assay kit (Beyotime) was employed to quantify the total protein concentration. Subsequently, the samples were subjected to electrophoresis by SDS-PAGE after which they were transferred onto PVDF membranes (Millipore, Billerica, MA). The membranes were blocked with 5% skim milk for 1.5 h and incubated with primary antibodies, including those against USP25 (1:1000; catalog no. ab187156; Abcam), ROCK1 (1:1000; catalog no. 21850-1-AP; Proteintech, China), ROCK2 (1:1000; catalog no. ab125025; Abcam), pNF-κB p65 (Ser536) (1:1000; catalog no. 93H1; CST), tNF-κB p65 (1:1000; catalog no. D14E12; CST), Iba-1 (1:1000; catalog no. ab178847; Abcam), TNF-α (1:1000; catalog no. 20291-1-Ig; Proteintech), IL-1β (1:1000; catalog no. ab254360; Abcam), MCP-1 (1:1000; catalog no. bs-1955R; Bioss, China), and β-actin (1:1000; catalog no. TA-09; ZSGB-BIO, China). Subsequently, the blots were subjected to incubation for 1.5 h with horseradish peroxidase-conjugated secondary antibodies. Thereafter, an enhanced chemiluminescence kit (29050, Engreen, Beijing, China) was employed to visualize the antibody reactivity as directed by the manufacturer.

### RNA Extraction and Quantitative Real-Time PCR Analysis

Trizol reagent (1 mL, Invitrogen) and 0.25 mL of chloroform were used to isolate total RNA from retinal tissue or cellular samples, followed by a 15-min incubation period. Subsequently, after subjecting the mixture to a 15-min centrifugation run at 12,000 g at 4 °C, an equivalent amount of isopropanol was introduced. After another centrifugation step at 12,000 g for 15 min at 4 °C, the RNA pellet was washed in 75% and 100% ethanol and re-suspended in 10–20 μL of RNase-free water Roche Premix (Roche) was used to reverse-transcribe 1 µg of total RNA for cDNA synthesis. The Fast SYBR Green Master Mix (Roche) was employed to conduct the qRT-PCR. The primer sequences that correspond to the target genes are presented in Table S1.

### Hematoxylin and Eosin (HE) Staining and Retinal Thickness Measurements

The eyes of mice were enucleated and fixed in a specialized eyeball fixative for 48 h. The entire eyeball was dehydrated using a series of graded ethanol solutions and subsequently embedded in paraffin. Next, the samples were sliced to a thickness of 5 μm and thereafter stained with HE following conventional procedures. Observations were conducted *via* light microscopy (Leica, Germany).

### Statistical Analysis

The data are presented as mean ± SEM and were subjected to statistical analysis using the two-tailed Student’s *t*-test (unpaired *t*-test) or one-way ANOVA, employing GraphPad Prism software (version 8.0.1, USA). A *P* value of < 0.05 was deemed statistically significant.

## RESULTS

### USP25 is Upregulated in STZ-Induced Diabetes in Wild-Type Mice

**Fig. 1:**
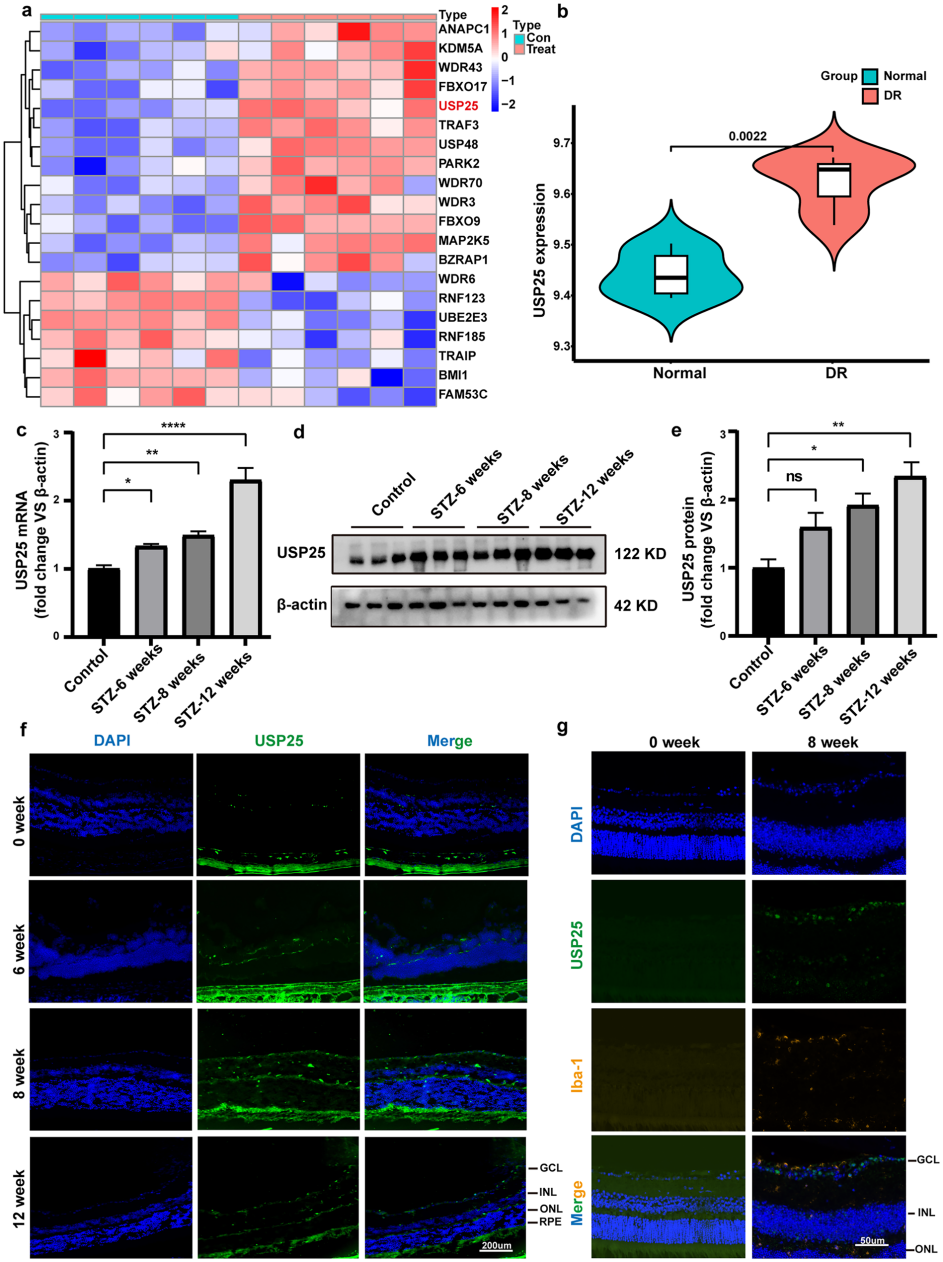
Expression level of USP25 is upregulated following diabetes in wild-type mice. **a** Heat map illustrating differentially expressed URGs in the GSE111465 dataset. **b** Elevated expression of USP25 in DR retinas compared to normal retinas. **c** The expression of USP25 was compared at 6, 8, or 12 weeks post-STZ treatment in diabetic and non-diabetic retinas using qRT-PCR assays (*P < 0.05, one-way ANOVA, *n* ≥ 3). **d**-**e** WB and quantitative analyses were conducted to assess USP25 expression levels in diabetic and non-diabetic retinas at 6, 8, or 12 weeks following diabetes induction (*P < 0.05, one-way ANOVA, *n* = 3). **f** Immunofluorescence staining of USP25 in various retinal layers at 6, 8, or 12 weeks post-diabetes induction. Representative images are displayed. Scale bar, 200 μm. **g** Immunofluorescence demonstrating co-expression of USP25 (green) with Iba-1 (gold), which indicates microglia. Scale bar, 50 μm.

Neuroinflammation has been identified as a critical key factor in both the initiation onset and progression of DR [17]. Abnormal expression levels of ubiquitination are also important in the development of DR [18, 19]. To elucidate the role of URGs in DR, we extracted URG data from the GSE111465 dataset in the GEO database. Subsequently, we performed a differential heat map analysis (Fig. 1a), which highlighted a significant difference in the expression of USP25. Subsequently, we conducted a detailed investigation of the upregulation of USP25 expression in the retinas of mice with STZ-induced diabetes for 6 weeks using raw matrix data (Fig. 1b). To assess the relationship between USP25 expression levels and STZ-induced DM, we established a DR animal model. After 6, 8, and 12 weeks of STZ-induced DM, the expression and distribution of USP25 were assessed using western blot (WB), immunofluorescence, and PCR techniques. As the duration of diabetes increased, USP25 expression gradually elevated, peaking at 12 weeks of STZ-induced DM (Fig. 1c-e). For further investigation, we initially analyzed the expression and distribution of USP25 protein over the course of DR through immunofluorescence. At 6 weeks of STZ-induced DM, USP25 was prominently concentrated in the outer nuclear layer, followed by a progressive increase as the disease advanced, extending from the outer to the inner nuclear layer and finally the ganglion cell layer (Fig. 1f). This pattern aligned with previously reported microglia expression patterns in STZ-induced DR [16]. Microglia, as resident immune cells in the retina, are often activated in degenerative and inflammatory retinal conditions, including DR. Therefore, we suspected that the heightened expression of USP25 may be associated with abnormal microglial function. Immunofluorescence analyses demonstrated an increase in the immunoreactivity of USP25 and Iba-1 in the retinas of mice after 8 weeks of STZ-induced DM. In comparison to the control group, the number and the regions of Iba-1-positive cells were significantly increased in the mice treated with STZ. Additionally, the fluorescence intensity of USP25 increased accordingly. USP25 was significantly colocalized with Iba-1. Through the IF of Iba-1 to identify microglial cells, we validated a limited overlap between USP25 (green) and Iba-1 (gold) signals in DR (Fig. 1g).

### USP25 Inhibition Ameliorates Retinal Inflammatory Response and Structural Damage

**Fig. 2:**
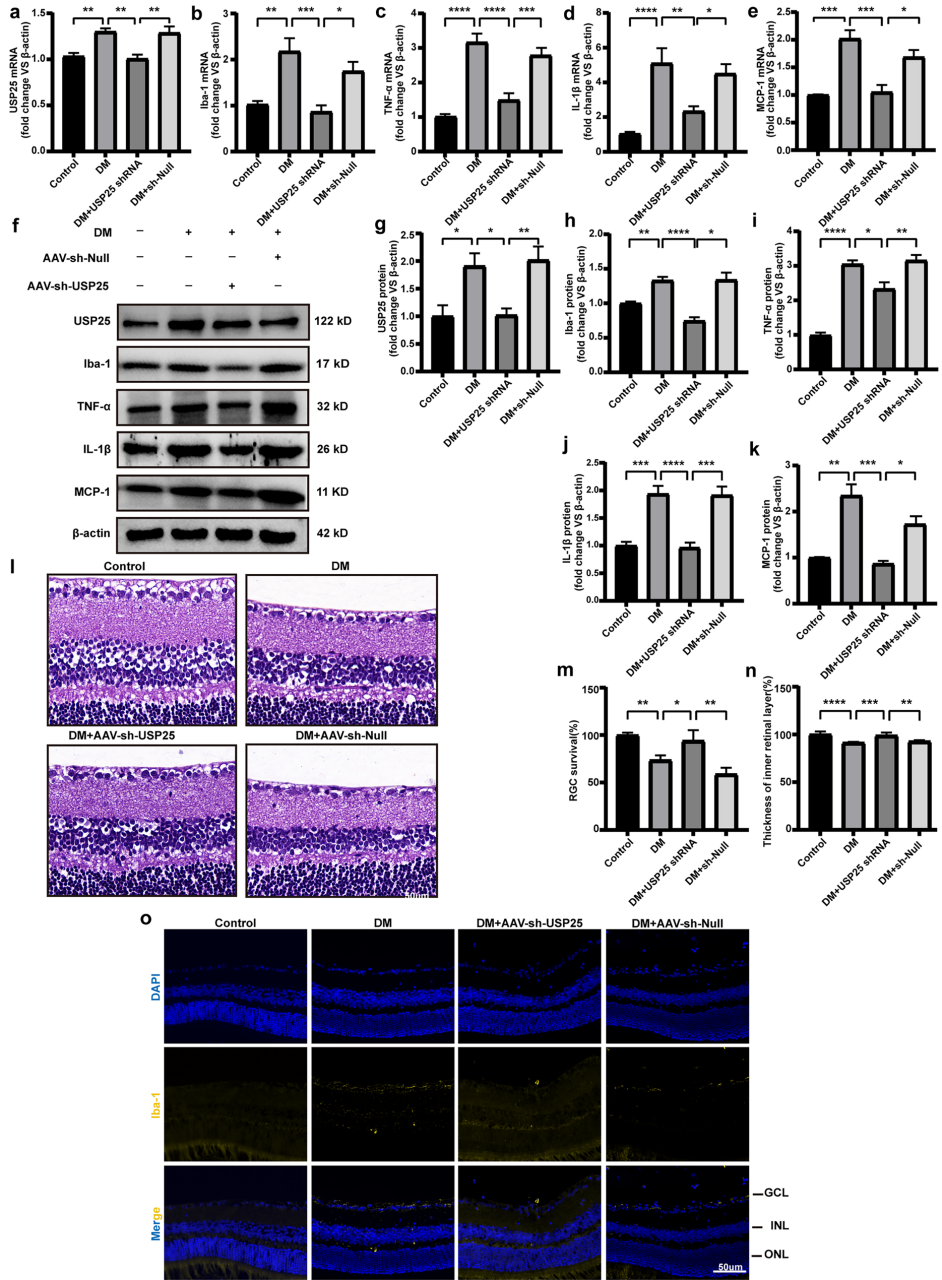
USP25 regulates retinal inflammatory responses induced by diabetes. **a**–**e** C57BL/6 J mice who developed diabetes due to STZ were injected intravitreally with NC AAV-Null, AAV-sh-USP25, or were left untreated at 4 weeks post-diabetes induction. The Ctrl group consisted of non-diabetic C57BL/6 J mice. USP25, IL-1β, MCP-1, TNF-α, and Iba-1 expression levels were determined using qRT-PCR assays (*P < 0.05, one-way ANOVA, *n* ≥ 3). **f**–**k** WB and quantitative analyses were implemented to examine the USP25, TNF-α, IL-1β, MCP-1, and Iba-1 protein expression in retinal lysates (*P < 0.05, one-way ANOVA, *n* ≥ 3). **l**–**n** HE staining was performed to detect retinal thickness and ganglion cell apoptosis in the Ctrl group and mice with diabetes caused by STZ injected with NC AAV-Null or AAV-sh-USP25 following 4 weeks of diabetes induction. Representative images correspond to 8 weeks post-diabetes induction (*P < 0.05, one-way ANOVA, *n* ≥ 3). **o** Representative images of immunofluorescence staining of Iba-1 to detect retinal reactive microglia. Scale bar, 50 μm.

The persistent activation of microglia in the retina at locations where DR lesions occur can contribute to the continuation of chronic neuroinflammation [20, 21]. We next investigated whether USP25 regulates the diabetes-induced inflammation in the retina *in vivo*. We administered intravitreal injections of NC (AAV-sh-Null) and AAV-sh-USP25 to STZ-induced diabetic mice. The effectiveness of USP25 knockdown was assessed using qRT-PCR assays (Fig. 2a). We employed qRT-PCR and WB analysis to assess changes in the expression levels of inflammatory mediators. The results showed that intravitreal injection of AAV-sh-USP25 alleviated diabetes-induced retinal inflammation 8 weeks after STZ injection, as evidenced by reduced expression levels of Iba-1, MCP-1, IL-1β, and TNF-α (Fig. 2b–k). Furthermore, we performed HE staining to assess changes in retinal thickness and ganglion cell apoptosis. We found that, compared with the AAV-sh-Null group, USP25 inhibition led to increased cell count, thicker retinal layers, better cell alignment, and reduced retinal structural damage in the ganglion cell layer (Fig. 2l–n). Immunostaining assays revealed that intravitreal injection of AAV-sh-USP25, as opposed to AAV-sh-Null, alleviated retinal reactive gliosis, as indicated by reduced Iba-1 staining (Fig. 2o), and enhanced the survival of retinal ganglion cells (RGCs) (Fig. 2m). Collectively, these results illustrate the potential function of USP25 in contributing to the progression of DR by activating microglia and influencing the secretion of their inflammatory factors.

### Expression and Subcellular Localization of USP25 in Microglial Cell Lines

**Fig. 3:**
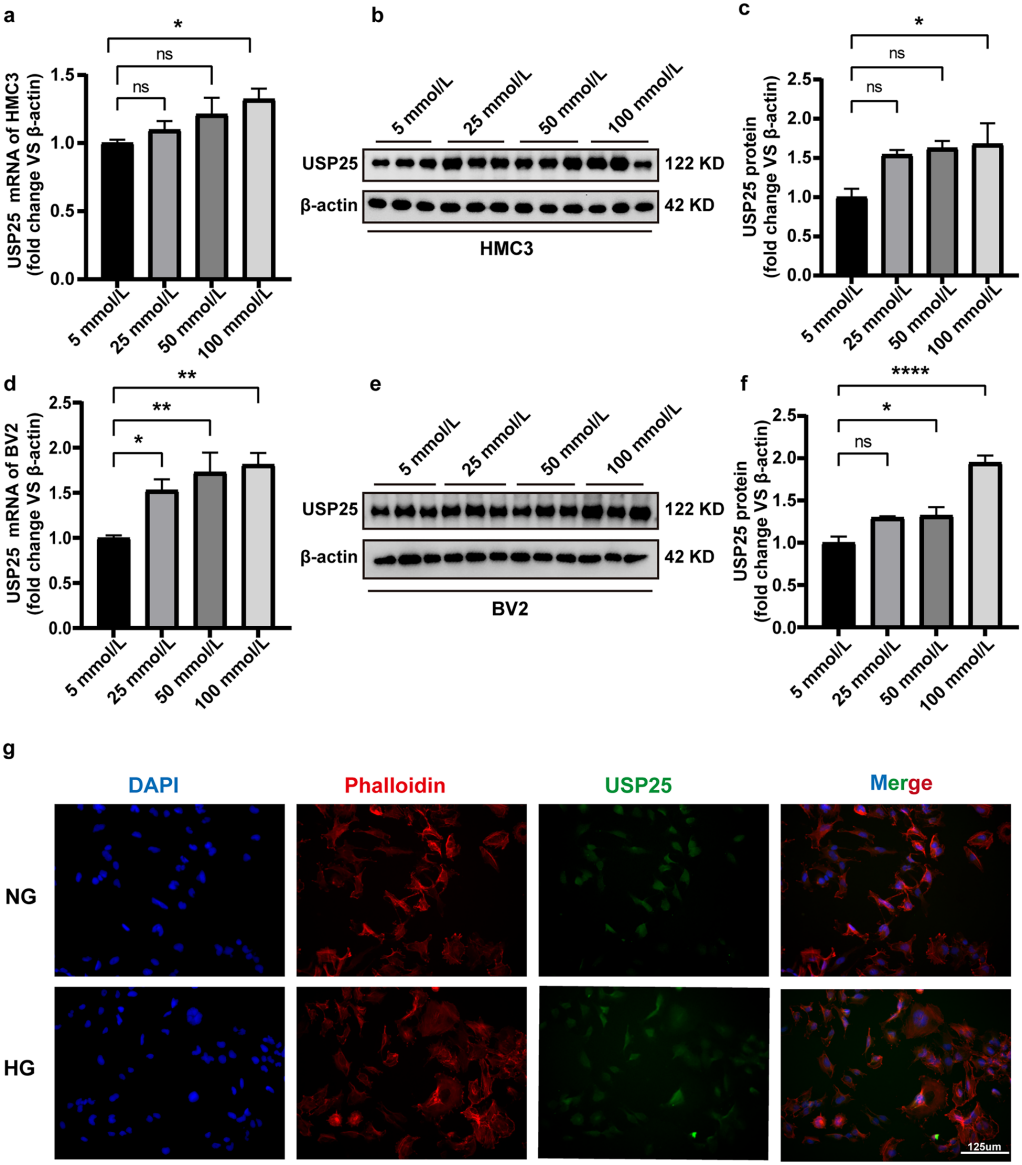
Expression of USP25 in microglia *in vitro*. **a** Comparative analysis of USP25 expression between normal glucose and HG conditions at 25, 50, or 100 mmol/L for 48 h in HMC3 cells was performed *via* qRT-PCR assays (*P < 0.05, one-way ANOVA, *n* ≥ 3). **b-c** WB and quantitative analyses were executed to determine the USP25 expression levels between normal glucose and HG conditions at 25, 50, or 100 mmol/L for 48 h in HMC3 cells (*P < 0.05, one-way ANOVA, *n* ≥ 3). **d** Comparative analysis of USP25 expression between normal glucose and HG conditions at 25, 50, or 100 mmol/L for 48 h in BV2 cells was conducted *via* qRT-PCR assays (*P < 0.05, one-way ANOVA, *n* ≥ 3). **e-f** WB and quantitative analyses were executed to determine the USP25 expression levels between normal glucose and HG conditions at 25, 50, or 100 mmol/L for 48 h in BV2 cells (*P < 0.05, one-way ANOVA, *n* ≥ 3). **g** Representative images of immunofluorescence staining of USP25 (red) or phalloidin (green) to detect subcellular localization of USP25. Scale bar, 125 μm.

Based on the aforementioned findings, we evaluated the possible function of USP25 in microglia by examining its expression pattern in response to diabetic stress *in vitro*. We utilized HMC3 cells exposed to high concentrations of glucose. We employed qRT-PCR and WB analysis to detect USP25 expression. USP25 expression levels were markedly upregulated in HMC3 cells following exposure to HG conditions (Fig. 3a–c). Similarly, we investigated USP25 expression in the BV2 cell line, where we observed a consistent pattern with that observed in HMC3 cells (Fig. 3d–f). USP25 expression increased concomitantly with the elevation of glucose concentration in both cell lines. Additionally, we conducted immunofluorescence analyses to investigate the subcellular localization of USP25 under these conditions in HMC3 cells. We found that USP25 was predominantly expressed in the cytoplasm during HG conditions, indicating its involvement in exerting its physiological functions (Fig. 3g).

### Inhibition of USP25 Improves Microglia Inflammatory Response

**Fig. 4:**
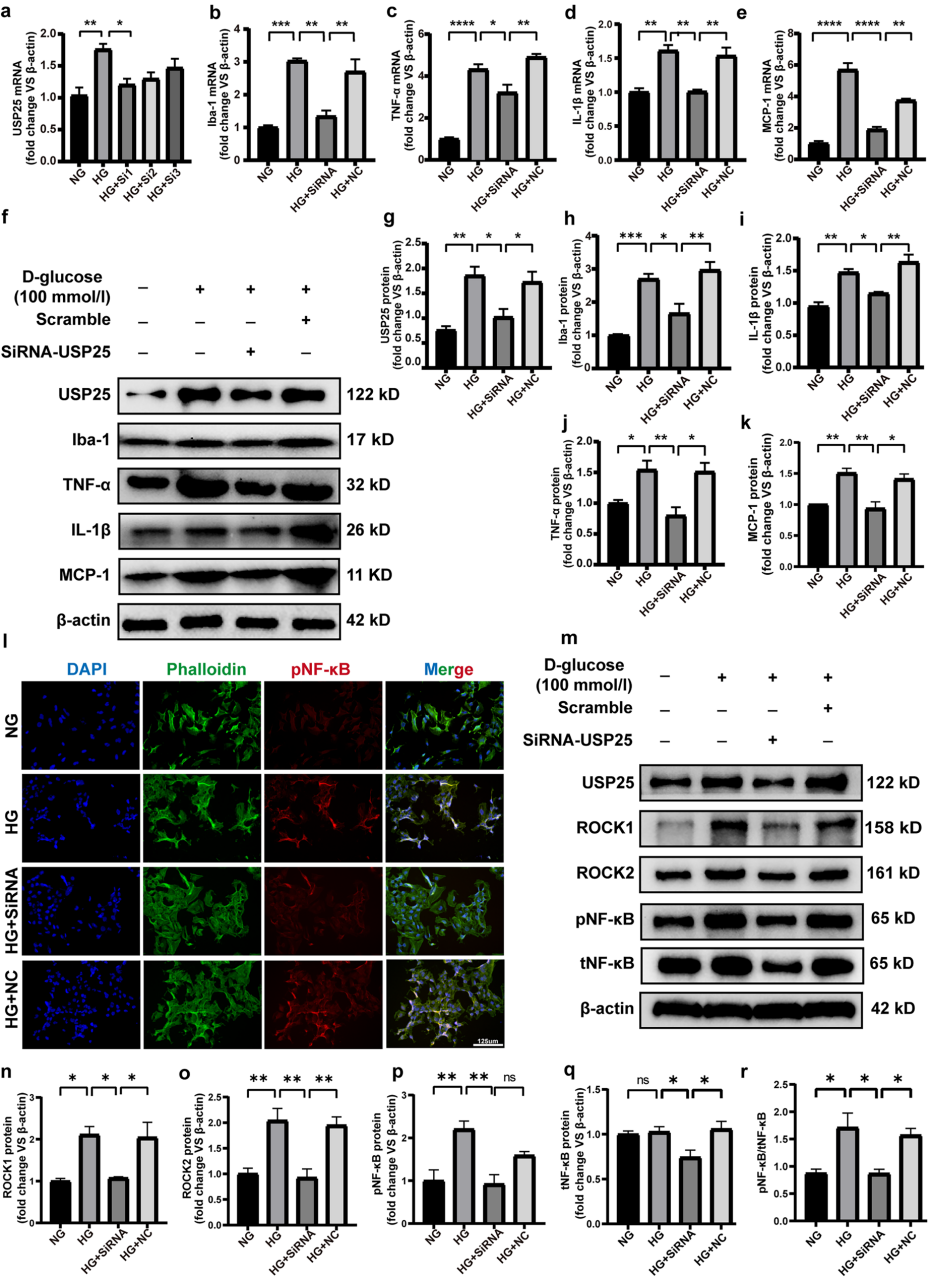
USP25 regulates microglia function after diabetic stress *in vitro*. **a** HMC3 cells were transfected with NC, USP25 siRNA, or with no treatment (Ctrl) for 48 h under HG conditions. The expression levels of USP25 were assessed *via* qRT-PCR (*P < 0.05, one-way ANOVA, *n* ≥ 3). **b**–**e** HMC3 cells were transfected with NC, USP25 siRNA, or with no treatment (Ctrl) for 48 h under HG conditions. TNF-a, IL-1β, MCP-1, and Iba-1 expression levels were determined using qRT-PCR assays (*P < 0.05, one-way ANOVA, *n* ≥ 3). **f**–**k** WB and quantitative analyses were conducted to assess USP25, TNF-a, MCP-1, IL-1β, and Iba-1 expression levels (*P < 0.05, one-way ANOVA, *n* ≥ 3). **l** Representative images of immunofluorescence staining of pNF-κB (red) and phalloidin (green) to detect subcellular localization of pNF-κB. Scale bar, 125 μm. **m**–**r** HMC3 cells following transfection with NC, USP25 siRNA, or with no treatment (Ctrl) for 48 h under HG conditions. WB and quantitative analyses were conducted to assess the ROCK1, ROCK2, pNF-κB, and tNF-κB expression levels (*P < 0.05, one-way ANOVA, *n* ≥ 3).

To further understand whether USP25 affects the inflammatory response of microglia, we knocked down USP25 expression in the HMC3 cell line. First, we selected one of the three siRNA sequences displaying the highest knockdown efficiency for subsequent experiments. qRT-PCR revealed that Si3 exerted a significant inhibitory effect (Fig. 4a). The results showed that increased expression of USP25 promoted microglia activation, thereby contributing to the augmentation of retinal inflammatory responses and vascular permeability. Conversely, the reduction in USP25 levels resulted in the reversal of these effects *in vitro* (Fig. 4b–k). However, the precise underlying mechanisms remain unclear. Our research group has been dedicated to the study of the ROCK signaling axis [22]. Neuroinflammation is a typical feature of DR [23]. The Rho/ROCK signaling pathway has been shown to activate central microglia, leading to the production of inflammatory markers (IL-1β, TNF-α), thereby promoting neuroinflammation [24, 25]. We found a positive correlation between USP25, ROCK1, and ROCK2 in the GSE111465 dataset (Fig. S1). Therefore, we investigated the expression of ROCK1, ROCK2, and NF-κB *via* WB. The results highlighted a reduction in the expression level of ROCK1, ROCK2, pNF-κB, and tNF-κB following USP25 inhibition (Fig. 4m–r). We further examined the nuclear translocation of pNF-κB and observed an increase in microglial nuclear translocation under HG conditions, which was subsequently reversed upon the inhibition of USP25 (Fig. 4l).

### ROCK Pathway Inhibitors Improve Microglia Inflammatory Response, Activation Induced by High Glucose

**Fig. 5:**
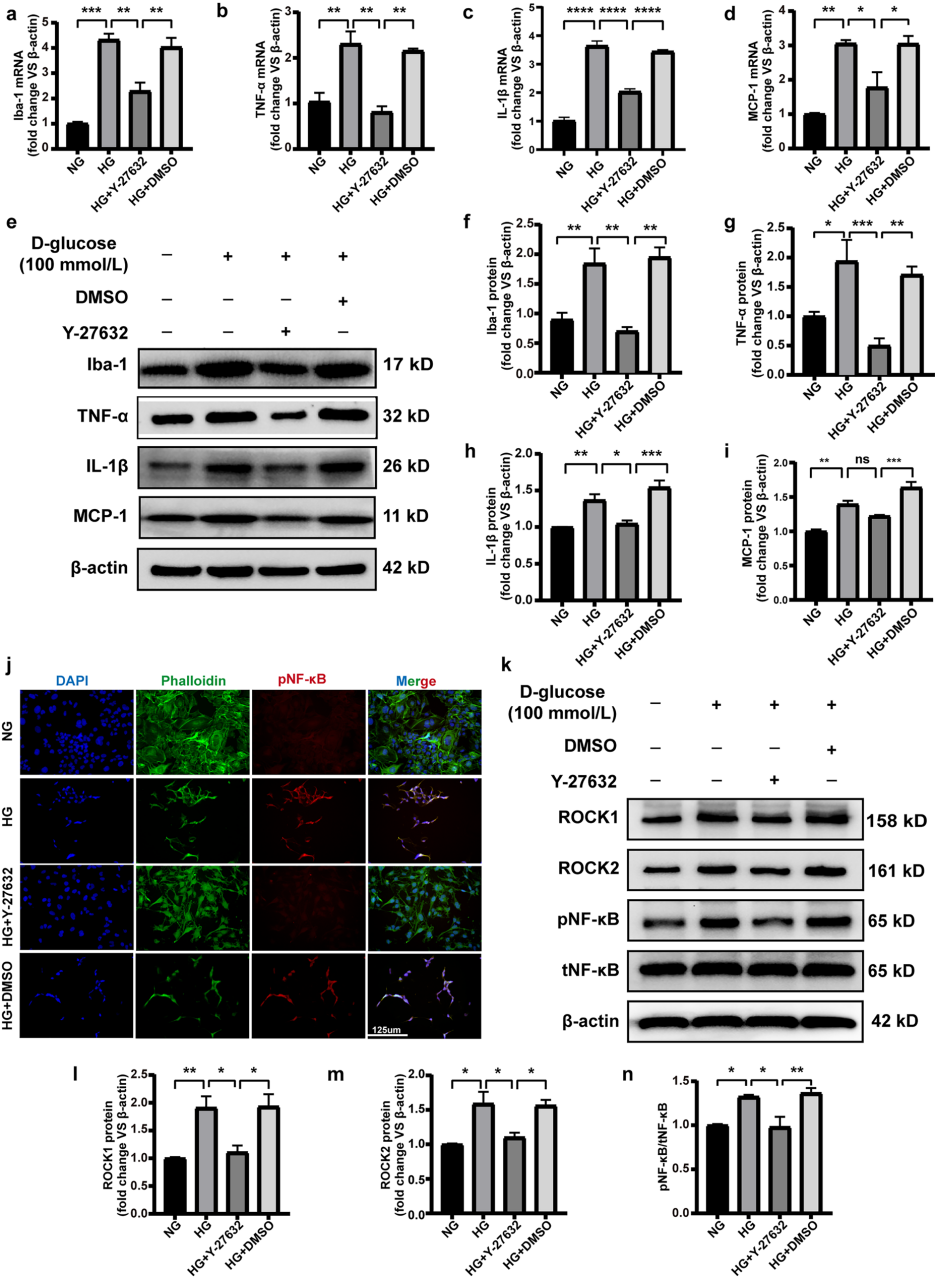
Y-27632 regulates microglia function after diabetic stress *in vitro*. **a**–**d** HMC3 cells were co-cultured with DMSO, Y-27632, or remained without treatment (Ctrl) for 48 h under HG conditions. IL-1β, MCP-1, TNF-α, and Iba-1 expression levels were determined by qRT-PCR (*P < 0.05, one-way ANOVA, *n* ≥ 3). **e**–**i** WB and quantitative analyses were performed to assess IL-1β, MCP-1, TNF-α, and Iba-1 expression levels (*P < 0.05, one-way ANOVA, *n* ≥ 3). **j** Representative images of immunofluorescence staining of pNF-κB (red) and phalloidin (green) to detect subcellular localization of pNF-κB. Scale bar, 125 μm. **k**–**n** WB and quantitative analyses were applied to determine the ROCK1, ROCK, pNF-κB, and tNF-κB protein expression levels in cell lysates (*P < 0.05, one-way ANOVA, *n* ≥ 3).

After silencing USP25, we observed a decrease in ROCK/NF-κB signaling. However, ROCK/NF-κB involvement in microglial cells under HG conditions has not been described in any previous research. To address this gap, we administered the Rho/ROCK inhibitor Y-27632 to investigate whether ROCK/NF-κB plays a role in HG-induced microglial activation. Following the administration of Y-27632, we conducted qRT-PCR and WB analyses to assess the expression profiles of MCP-1, IL-1β, Iba-1, and TNF-α. We observed a reduction in the levels of the HG-induced microglial inflammatory factors TNF-α and IL-1β, as well as a decrease in the activation and expression of chemokines Iba-1 and MCP-1 (Fig. 5a–i). Furthermore, we examined the nuclear translocation of pNF-κB after Y-27632 administration. We noted a decrease in nuclear translocation, consistent with the findings observed after inhibiting USP25 (Fig. 5j). We additionally examined the expression levels of ROCK and NF-κB. In HG conditions, pNF-κB expression was upregulated but subsequently decreased after Y-27632 administration, whereas the expression of tNF-κB remained essentially unchanged (Fig. 5k–n). These findings collectively suggest that Y-27632 mitigates HG-induced microglial activation and the secretion of inflammatory factors by downregulating the NF-κB pathway activation.

### *In Vivo* Validation of USP25’s Role in Regulating the Expression of the ROCK/NF-κB Signaling Axis

**Fig. 6:**
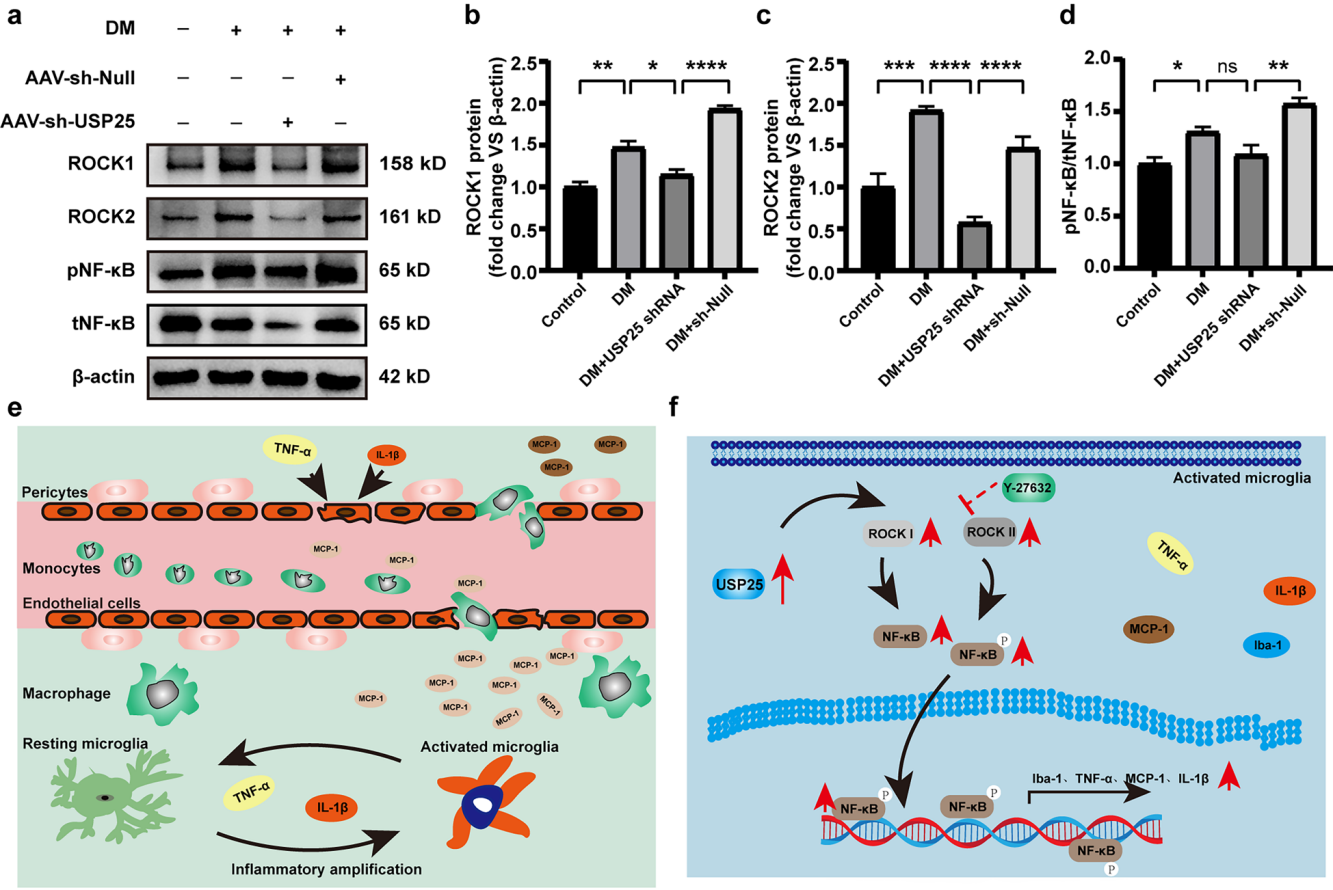
USP25-ROCK1/ROCK2-NF-κB signaling axis regulates retinal inflammatory responses *in vivo*. **a**–**d** C57BL/6J mice who developed diabetes upon exposure to STZ were injected intravitreally with NC AAV-Null, AAV-sh-USP25, or were left untreated at 4 weeks post-diabetes induction. The Ctrl group contained non-diabetic C57BL/6J mice. WB and quantitative analyses were performed to examine the ROCK1, ROCK, pNF-κB, and tNF-κB protein expression in retinal lysates (*P < 0.05, one-way ANOVA, *n* ≥ 3). **e** Schematic diagram of microglia involved in the development of DR. **f** A schematic diagram illustrating the involvement of USP25 in the development of DR through the regulation of the ROCK/NF-κB signaling pathway.

To further explore the impact of USP25 on microglia activation *via* the ROCK/NF-κB signaling axis *in vivo*, we examined the expression levels of ROCK1, ROCK2, and NF-κB within the retina. We found that, compared with the AAV-sh-Null group, USP25 inhibition can reduce in the expression of ROCK/NF-κB, aligning with our earlier *in vitro* findings (Fig. 6a–d). Therefore, USP25 fosters the transcription and activation of inflammatory genes in microglia by modulating the ROCK signaling axis, leading to downstream NF-κB activation.

## DISCUSSION

In most countries, DR stands out as the most prevalent complication of DM, persistently ranking as a major contributor to vision loss worldwide. Extensive research spanning half a century has explored its etiology and pathology, yet disappointingly, there remains a scarcity of viable therapeutic options [26]. Although some novel treatments, such as new steroids and intravitreal vascular endothelial growth factor inhibitors (“anti-VEGFs”), a substantial proportion of patients, up to 50%, do not respond satisfactorily. Furthermore, laser photocoagulation continues to serve as a primary therapy for individuals with proliferative DR, despite its inherently destructive nature [27]. Thus, while significant progress has been made, developing robust and reliable methods to intervene effectively before visual loss occurs necessitates fundamental mechanisms understanding.

Microglia play a pivotal role in initiating and exacerbating retinal inflammatory responses, leading to the disruption of retinal vascular barrier function and neuronal apoptosis (Fig. 6e). The activation of microglia in DR has been extensively investigated and shown to occur before vascular lesion formation. Cellular damage and Amadori-glycated albumin are two of the many stimuli that might activate microglia [28]. Activated microglia can disrupt the microenvironment’s homeostasis and inflict damage on neurons or vascular cells [21]. Suppression of inflammation instigated by microglia has been shown to offer prophylactic benefits against DR. However, the precise molecular mechanisms underlying microglia-driven neuroinflammation remain incompletely understood. Here, we show that diabetic stress causes a dramatic upregulation of the ubiquitination-associated protein USP25. Notably, preserving visual function and visually directed behaviors, protecting RGCs from diabetic damage, suppressing retinal reactive gliosis, and slowing the retinal inflammatory response may be achieved by inactivating USP25-mediated signaling. Altogether, our findings illustrate that USP25 could act as a useful target for neurovascular protection and vision preservation.

USP25 has been implicated in the activation of microglia within the central nervous system (CNS) [10]. In addition, elevated USP25 expression promotes inflammation and fibrosis in diabetic nephropathy [11]. Furthermore, USP25 is instrumental in the regulation of immune responses and is a recognized member of the deubiquitinating enzyme family. Initially identified as a negative regulator of IL-17-mediated signaling and inflammatory responses, USP25 plays a significant role in autoimmune and infectious processes [29]. Earlier research has revealed that USP25 restrains HBO1 ubiquitination, leading to increased HBO1 stability, which in turn results in the accumulation of HBO1 and subsequently augments HBO1-mediated transcription of inflammatory genes [30]. Moreover, it also enhances the stability of the amyloid precursor protein, elevating protein expression levels and ultimately raising β-amyloid levels, thereby contributing to its accumulation in the brain and promoting the development of Alzheimer’s disease [31]. In our study, we observed that reduced USP25 expression inhibited the development of retinal inflammatory responses, suppressed ganglion cell apoptosis, and reduced retinal damage in a hyperglycemic environment. Throughout the course of DR, an increased expression of USP25 was notably observed in microglia in comparison to other retinal cells. Intravitreal injections with AAV-sh-USP25 demonstrated an anti-inflammatory effect in microglia, as evidenced by reduced expression of the microglia activation marker Iba-1 and pro-inflammatory markers IL-1β, MCP-1, and TNF-α (Fig. 2b–k). Furthermore, intravitreal injection of AAV-sh-USP25 mitigated retinal reactive inflammation and enhanced the survival of RGCs (Fig. 2l–n). Hence, the activation of retinal microglia mediated by USP25 stands out as a critical step in the onset of DR.

In this study, the interactions among USP25, ROCK1, and ROCK2 were validated using bioinformatics (Fig. S1), immunoblotting, and immunofluorescence techniques. We calculated the correlation between USP25 and ROCK1, ROCK2 using Spearman correlation analysis and found that USP25 was positively correlated with the expression of ROCK1, ROCK2. RhoA/ROCK signaling induces alterations in the actin cytoskeleton, leading to cell migration, modulation of cellular motor properties, and activation of immune cells [32]. There are two primary intracellular ROCK isoforms, namely ROCK I and ROCK II, with ROCK II being predominantly expressed in the nervous system. Multiple CNS diseases have been associated with aberrant RhoA/ROCK pathway activation [33]. ROCKs have emerged as pivotal mediators of neuroinflammation and have a significant role in microglia activation [24]. Inhibition of the ROCK pathway has been shown to mitigate the inflammatory response in microglia. Fasudil reduces the nonspecific inflammatory response induced by AGEs in microglia and exerts its anti-inflammatory effects by suppressing the RhoA/ROCK signaling pathway, thereby reducing the nuclear translocation of NLRP3 and NF-κB [34]. In addition, recent studies have also demonstrated that melatonin treatment, when administered to a CNV model, reduces Rho/ROCK activity. Inhibition of the Rho/ROCK signaling axis, originally associated with promoting the M2 macrophage phenotype, induces polarization towards M1 macrophages and enhances the production of inflammatory markers, such as TNF-α [35]. In this case, we detected changes in the expression of ROCK1, ROCK2, pNF-κB, and tNF-κB after administration of USP25 SiRNA using WB. We found that USP25 reduced the expression of not only pNF-κB but also tNF-κB (Fig. 4m–r). This is consistent with the results *in vivo* (Fig. 6a–d). Furthermore, Rho/ROCK signaling is involved in central microglial cell polarization. In an ischemic and BV2 oxygen-deprivation cell model, the expression of RhoA and ROCK2 was upregulated. However, inhibiting Rho A and ROCK2 effectively suppressed ischemia-induced inflammatory responses in microglia, resulting in reduced microglial activation and decreased secretion of inflammatory factors [36]. To our surprise, in our study, the administration of the ROCK pathway inhibitor Y-27632 resulted in reduced secretion of inflammatory factors and chemokine expression in HG-induced microglial cells. This is the first time that we have found that Y-27632 also suppresses retinal inflammatory responses by inhibiting the expression of ROCK in microglia (Fig. 5a–i). Research has established that nonspecific inflammatory responses in microglia, triggered by AGE stimulation, primarily involve the RhoA/ROCK signaling pathway activation and a decrease in the nuclear translocation of NLRP3 and NF-κB, leading to pro-inflammatory effects [34]. We also observed a decrease in NF-κB expression and reduced nuclear translocation following Y-27632 administration (Fig. 5j). However, we noted a similar reduction in tNF-κB expression after silencing USP25, suggesting that USP25 may regulate NF-κB expression and activation through mechanisms other than its impact on ROCK pathway-mediated NF-κB phosphorylation levels. These alternative mechanisms will be the focus of our future investigations. Our study presents the novel finding that the USP25/ROCK axis modulates the hyperglycemia-mediated microglial inflammatory response, both *in vivo* and *in vitro*. Thus, the USP25-ROCK/NF-κB axis is closely associated with retinal neuroinflammation.

Our study has certain limitations. Although we clarified the existence of a regulatory relationship between USP25 and ROCK/NF-κB, the precise manner in which USP25 impacts the ROCK pathway remains unknown. We did not detect ubiquitination levels in microglia or retina after silencing USP25, and so we were unable to determine whether USP25 is dependent on its deubiquitination function to regulate ROCK1/ROCK2 expression. In addition, USP25 not only influences the expression of pNF-κB but also affects the expression of tNF-κB, which diverges from the ROCK pathway’s role in modulating the inflammatory response induced by HG in microglia. In future investigations, we intend to further elucidate these mechanisms, enhance our understanding of USP25’s role in DR, and explore potential pharmacological targets.

In conclusion, our research not only elucidates the regulatory mechanisms of ubiquitination in DR but also offers valuable insights into unresolved queries. These inquiries encompass the identification of molecules acting as sensors for diabetic neuroinflammation and the comprehension of molecules that can jointly regulate both retinal vascular dysfunction and neurodegeneration. Overall, the significant improvements in neuronal survival and reduction of retinal inflammatory responses observed in retinas lacking USP25 suggest that targeting USP25 holds promise as an effective strategy for managing retinal neuropathy. Subsequent efforts could center on the development of pharmaceutical agents that selectively target USP25-mediated signaling pathways and further clarify their roles in treating ocular neurovascular disorders. Collectively, our findings suggest that USP25 can trigger the ROCK signaling pathway, leading to the phosphorylation of NF-κB, heightened microglial activation, increased secretion of inflammatory factors, and an augmented retinal inflammatory response (Fig. 6f).

### Supplementary Information

Below is the link to the electronic supplementary material.

Supplementary file1 (DOCX 23 KB)

## Data Availability

All data generated and/or analyzed during the current study are included in this published article and its supplementary information files, or available from the corresponding authors on reasonable request.
