# Supplementary material for: A New Modulator of Neuroinflammation in Diabetic Retinopathy: USP25
Source: Inflammation. 2024 Mar 4;47(4):1520–35. doi: 10.1007/s10753-024-01991-x (PMC11343827; doi:10.1007/s10753-024-01991-x)

| **Table 1. The primer information** | |
| --- | --- |
| Human ACTB-Forward | CAGATGTGGATCAGCAAGCAGGAG |
| Human ACTB-Reverse | CGCAACTAAGTCATAGTCCGCCTAG |
| Human USP25-Forward | GTTGGAATATGCAAGGTTGGTT |
| Human USP25- Reverse | TGCCTGGTTCTGGATAAAGTAG |
| Human Iba-1-Forward | GAAGACTGGTGGGAGAGAAGGAGAG |
| Human Iba-1-Reverse | GCTTGTTGATCTCATCCAGCCTCTC |
| Human TNF-α-Forward | AAGGACACCATGAGCACTGAAAGC |
| Human TNF-α-Reverse | AGGAAGGAGAAGAGGCTGAGGAAC |
| Human IL-1β-Forward | GGACAGGATATGGAGCAACAAGTGG |
| Human IL-1β-Reverse | TCATCTTTCAACACGCAGGACAGG |
| Human MCP-1-Forward | GGCTGAGACTAACCCAGAAACATCC |
| Human MCP-1-Reverse | CTATGAGCAGCAGGCACAGAAGG |
| Mouse ACTB-Forward | TTCAAGCTGAACAAGACAGAGCT |
| Mouse ACTB-Reverse | AGTACTCCTGGAAGTCAACTTCA |
| Mouse USP25-Forward | AACCCTTCACTCAGTCTCGGATACC |
| Mouse USP25-Reverse | CTCTCCAGCACGCACAGTTCTTC |
| Mouse Iba-1-Forward | ATTATGTCCTTGAAGCGAATGC |
| Mouse Iba-1-Reverse | TCTCAAGATGGCAGATCTCTTG |
| Mouse TNF-α-Forward | ATGTCTCAGCCTCTTCTCATTC |
| Mouse TNF-α-Reverse | GCTTGTCACTCGAATTTTGAGA |
| Mouse IL-1β-Forward | CACTACAGGCTCCGAGATGAACAAC |
| Mouse IL-1β-Reverse | TGTCGTTGCTTGGTTCTCCTTGTAC |
| Mouse MCP-1-Forward | TTTTTGTCACCAAGCTCAAGAG |
| Mouse MCP-1-Reverse | TTCTGATCTCATTTGGTTCCGA |


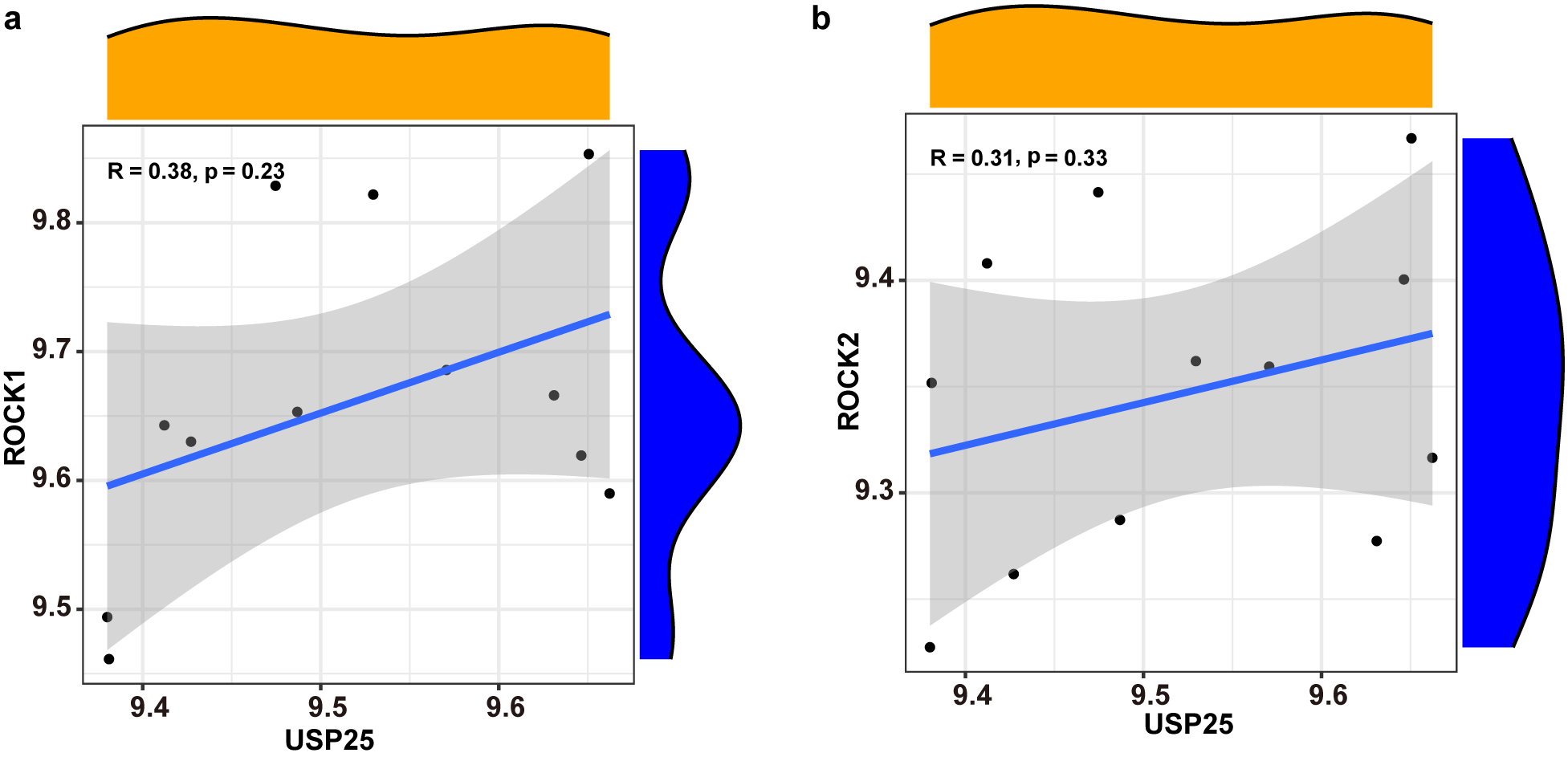

Supplement: Supplementary file 1 — Supplementary file1 (DOCX 23 KB) [file 10753_2024_1991_MOESM1_ESM.docx]
